# Pathophysiology, incidence, management, and consequences of cardiac arrhythmia in pulmonary arterial hypertension and chronic thromboembolic pulmonary hypertension

**DOI:** 10.1177/2045894019834890

**Published:** 2019-03-08

**Authors:** Meghan M. Cirulis, John J. Ryan, Stephen L. Archer

**Affiliations:** 1 Division of Pulmonary Medicine, Department of Medicine, University of Utah, Salt Lake City, UT, USA; 2 Division of Cardiovascular Medicine, Department of Medicine, University of Utah, Salt Lake City, UT, USA; 3 Department of Medicine, Queen's University, Kingston, ON, Canada

**Keywords:** sudden death, prognosis, survival, atrial fibrillation, supraventricular atrial arrhythmia, atrial flutter, right heart failure

## Abstract

Arrhythmias are increasingly recognized as serious, end-stage complications of pre-capillary pulmonary hypertension, including pulmonary arterial hypertension (PAH) and chronic thromboembolic pulmonary hypertension (CTEPH). Although arrhythmias contribute to symptoms, morbidity, in-hospital mortality, and possibly sudden death in PAH/CTEPH, there remains a paucity of epidemiologic, pathophysiologic, and outcome data to guide management of these patients. This review summarizes the most current evidence on the topic: from the molecular mechanisms driving arrhythmia in the hypertrophied or failing right heart, to the clinical aspects of epidemiology, diagnosis, and management.

## Introduction

Pulmonary hypertension (PH) is defined as an increase in mean pulmonary artery pressure (mPAP) to ≥25 mmHg at rest as assessed by right heart catheterization (RHC). As outlined in the most recent European Society of Cardiology (ESC)/European Respiratory Society (ERS) guidelines, PH is subdivided into groups 1–5: group 1 (pulmonary artery hypertension [PAH] including idiopathic, heritable, toxin-induced, or associated with other conditions); group 2 (PH due to left heart disease); group 3 (PH due to lung diseases and/or hypoxia); group 4 (chronic thromboembolic PH [CTEPH]); and, lastly, group 5 (PH with unclear or multifactorial mechanisms).^1^ Estimated five-year survival with PH is in the range of 38–59%,^2^ depending on the underlying etiology, with group 3 PH currently having the worst prognosis.^3^ In a recent population-based epidemiologic study of PH patients, a diagnosis of any form of PH was associated with a sevenfold increase in standardized mortality rate.^4^

The primary cause of death in PAH is thought to be right heart failure, occurring as a direct consequence of elevated PAP, although in some studies approximately 50% of patients died from another cause, with PH as a contributing factor.^5–7^ Arrhythmias, such as sinus tachycardia, atrial tachycardia, atrial fibrillation (AF), atrial flutter (Afl), sinus bradycardia, ventricular tachycardia (VT), and ventricular fibrillation (VF), have been recognized as serious, end-stage complications of PAH and CTEPH.^8^ Despite evidence that these arrhythmias contribute to symptom burden, morbidity, in-hospital mortality, and possibly sudden death,^8–12^ there remains scant data regarding the epidemiology, pathophysiology, and outcome of PAH patients with arrhythmia.

In this review, we explore the maladaptive and arrhythmogenic response of the right heart to group 1 and group 4 PH. We discuss the current patterns of clinical management, noting where these are evidence-based, and consider options for management of arrhythmia in PH. We also identify knowledge gaps and propose future directions.

While arrhythmia has been identified to coexist in all subgroups of PH, we will focus mainly on group 1 PH (PAH) and CTEPH in this review for two reasons. First, the majority of basic science studies pertaining to this topic have been conducted in PAH animal models and most clinical studies have predominantly included patients with PAH (group 1) and/or CTEPH (group 4). Second, the other subgroups (notably groups 2 and 3) have distinct etiology and pathophysiology, and therefore likely have differences in the mechanism of arrhythmogenesis, types of arrhythmia, and in the incidence and outcomes of these arrhythmias. For clarity and simplicity of text, we will refer to the group 1 (PAH) and group 4 (CTEPH) patients collectively as PAH/CTEPH, unless otherwise noted.

## The arrhythmogenic substrate of the right heart in pulmonary hypertension

A number of potential mechanisms have been identified as contributing to arrhythmia susceptibility in patients with elevated PAPs and pressure- and volume-overloaded right atrium and ventricle. One of the earliest studies noted vascular degeneration and infarction in the sinus and AV node and sudden death in patients with what was then called primary PH (now referred to as idiopathic PAH [IPAH]).^12^ In subsequent decades, a more granular mechanistic exploration has unfolded, revealing complex alterations in structure, electrophysiology, metabolism, and signaling pathways in the right heart.

### Autonomic nervous system

The autonomic nervous system plays a key role in the development and progression of PAH and right heart failure^13^ and has been implicated in pathogenesis of arrhythmia and sudden cardiac death (SCD).^14^ Sympathetic overdrive in PAH is manifested by decreased heart rate variability, a blunted baroreflex, and poor exercise capacity, and is associated with associated with worse clinical status and prognosis.^15–17^ Increased sympathetic activity has also been correlated with premature ventricular contractions and ventricular arrhythmia in PAH patients.^18^ Iodine-123-metaiodobenzylguanidine (123I-*m*IBG) myocardial imaging, a technique used to evaluate cardiac sympathetic nervous activity using single-photon emission computed tomography (SPECT), supports the aforementioned findings. Uptake of ^123^I-*m*IBG, a stable, modified form of guanethidine, occurs via the uptake-1 mechanism that normally uptakes norepinephrine.^19^ By comparing activity at 3-h scans to those at 30 min, one can assess washout of the mIBG, which is a measure of the retained NE within sympathetic neurons. When the sympathetic system is activated there is a reduction of pre-synaptic norepinephrine uptake, manifest as lower retention of *m*IBG. A low heart to mediastinal (HMR) ratio of *m*IBG (≤ 1.2) in late images predicts event-free survival in left heart failure.^20^ Increasing mPAP correlates with decreased mIBG activity in the right ventricle (RV), indicative of increased RV sympathetic activity. This decreased mIBG activity is associated with worse cumulative survival in PAH patients.^21–23^ Additionally, there is evidence of adrenergic remodeling in the RV, including downregulation and desensitization of β1-adrenergic receptors, as well as downregulation of α–adrenergic and dopaminergic receptors.^24,25^ In PAH patients with RV failure, there is also decreased adenylyl cyclase responsiveness to β-agonists and depletion of norepinephrine,^26,27^ with a loss of the ability to augment catecholamine levels with exercise.^28^

The intrinsic and extrinsic cardiac nervous systems have been implicated in the susceptibility to supraventricular arrhythmia (SVA) in PAH. In a recent study, Huang et al. studied atrial arrhythmia using a canine model in which PAH is induced in beagles using a single injection of 2 mg/kg of dehydromonocrotaline followed by an eight-week interval of observation while PAH develops.^29^ The dogs with PAH were more vulnerable than controls to induced AF/AFl. Histopathology demonstrated increased densities of sympathetic nerves and β-1 autonomic receptors in the right atrium, in contrast to the downregulation of β-adrenergic receptors in the failing RV.^25,27^ By systematically ablating the ganglionic plexi, which contain cholinergic and adrenergic neurons affecting the atrial myocardium, this reduced susceptibility to atrial arrhythmia, thereby demonstrating a pivotal role for the adrenergic component of the autonomic nervous system in regulating vulnerability to atrial arrhythmia in PAH.

### Electrical remodeling

Stretch and fibrosis of the right atrium constitute an arrhythmogenic substrate, which combined with electrical remodeling due to changes in expression and function of ion channels in cardiomyocytes, increases susceptibility to and propagation of arrhythmia.^30–33^ An electrophysiology study comparing the right atrium of eight patients with longstanding IPAH versus 16 matched controls demonstrated prolongation of sinus node recovery time and reduction in tissue voltage with electrically silent areas in the atrium. There was also slowing of atrial conduction and activation times and an increase in areas of complex fractionated activity (critical sites for AF perpetuation).^34,35^ These factors are associated with increased susceptibility to induction AF, as stimulated by either increased automaticity (ectopy) or single and multiple re-entrant pathways.^30,36^

Right ventricular hypertrophy (RVH) and fibrosis are seen in patients with PAH/CTEPH and are associated with increased all-cause mortality.^37^ Downregulation of inward rectifying potassium channels and slowed ventricular conduction (secondary to decreased gap junction expression, specifically connexin-43 remodeling, and fibrosis) impairs depolarization and repolarization in RVH.^38^ Similar changes also cause arrhythmogenic early and late after- depolarizations and increase susceptibility to spontaneous or induced ventricular arrhythmia in models of RVH and left ventricular hypertrophy (LVH).^39–41^ Changes in Ca^2+^ handling has also been implicated in the prolongation and dispersion of action potential duration in rat models of PH.^42,43^ In aggregate, these active and passive changes in the electrical system in the RV predispose to creation of reentrant circuits and arrhythmia.^44,45^ Structural remodeling, for instance myocyte fiber angle disarray, can also change electrical conduction leading to prolonged action potential duration and arrhythmia vulnerability, as seen in the monocrotaline-PAH rat model.^46^

### Ischemia

Ischemia is a known pro-arrhythmic factor in the left ventricle;^47,48^ likewise, RV ischemia is thought to contribute to arrhythmia vulnerability in PAH/CTEPH. Elevated troponin levels in PAH patients reflect this ischemia and are associated with increased mortality, although not specifically with arrhythmia.^49^ The genesis of ischemia in RVH is multifactorial, reflecting some combination of changes in epicardial coronary artery perfusion pressure (reduced perfusion pressure in the right coronary artery due to elevated right ventricular systolic pressure [RVSP],^50^ left main coronary artery compression^51^) and microvascular abnormalities (impaired angiogenesis manifest as capillary rarefaction related to decreased angiogenic gene expression).^52–54^ Secondary ischemia-related metabolic changes in the maladaptive RV, such as induction of increased rates of uncoupled glycolysis, are thought to perpetuate the vicious cycle of ischemia.^54^

## Electrocardiogram (ECG) abnormalities

Changes in the ECG are common in patients with PAH/CTEPH; however, there is controversy regarding the clinical significance of these abnormalities, and therefore uncertainty regarding the utility of screening electrocardiography. Kanemoto et al.^11^ reviewed 171 ECGs of 101 patients with PAH and found “arrhythmia” in 17.8% of all cases, with a higher prevalence (33.9%) among the deceased cohort. Three abnormalities accounted for 70% of all observed arrhythmias: sinus tachycardia (38%); sinus bradycardia (18%); and first-degree A-V block (15%); with sinus tachycardia being significantly associated with mortality. A more contemporary investigation examined the progression of ECG changes in 50 PAH patients and found a significant increase in median heart rate, PR interval, QRS duration, R/S ratio in lead V_1_, and QTc duration in the ECG recorded closest to death, compared to the ECG recorded at the time of initial diagnosis. No PAH patient retained a normal ECG close to death.^55^

As previously discussed, sinus tachycardia likely reflects autonomic dysregulation.^15^ In addition, in patients with RV failure and PH, stroke volume may be fixed (and low) and sinus tachycardia is likely a reactive mechanism to maintain cardiac output. Loss of heart rate variability is a marker of autonomic dysfunction that reflects tonic activation of the autonomic nervous system and may also be a homeostatic attempt to maintain cardiac output. Heart rate variability is blunted during exercise testing of PAH patients; a decrease in heart rate variability at rest parallels the severity of PH in PAH, as measured by mPAP.^17,56^ Sinus tachycardia and decreased heart rate variability may be useful predictors of susceptibility to SVA.

In left heart disease and other non-cardiac conditions, prolongation of the QT interval on ECG is described as a precursor to ventricular arrhythmia and is associated with increased mortality and sudden death.^8,57–64^ In PAH, longer QTc and QTc dispersion are correlated with increasing mPAP.^65^ In an age- and sex-matched cohort study of patients receiving PAH-specific therapy, both QRS duration and QTc interval were prolonged in 202 PAH patients versus 100 controls. A QTc duration ≥480 ms was an independent predictor of mortality, even in the subgroup of PAH and CTEPH.^66^ There was no difference in serum potassium to explain the prolongation of QTc, as might be expected with aggressive diuretic use. QTc prolongation correlated with impaired RV function, increased RV mass (measured by cardiac MRI), and poor prognosis.

In animal models of PAH, QT prolongation is associated with longer action potential duration and a proarrhythmic state.^43^ In the monocrotaline-rodent PAH model, QT prolongation is associated with decreased expression of repolarizing, voltage-gated (Kv) potassium channels (Kv1.2, Kv1.5, and Kv4.2) in the RV myocytes and is reversed with metabolic therapies, such as the pyruvate dehydrogenase kinase inhibitor, dichloroacetate.^38^ The etiology of prolonged QTc in patients with PH has not been clearly defined but may be a result of chronic ischemia in RVH and/or changes in the sympathetic nervous system, as discussed previously.^38,67^ Extrapolation from other disease processes would suggest an that such ionic remodeling and QTc prolongation would increase susceptibility to ventricular arrhythmia; however, this has not been directly studied in PAH/CTEPH patients and presents an opportunity for future investigation.

QRS prolongation has also been an area of active study in PAH. QRS duration has been found to be longer in patients with PH compared to controls and is associated with indices of decreased RV function.^66^ More specifically, prolonged QRS duration can contribute to RV dysynchrony, which is frequent in PAH and is an independent predictor of clinical worsening.^68,69^

## Supraventricular arrhythmias

**Fig. 1.**
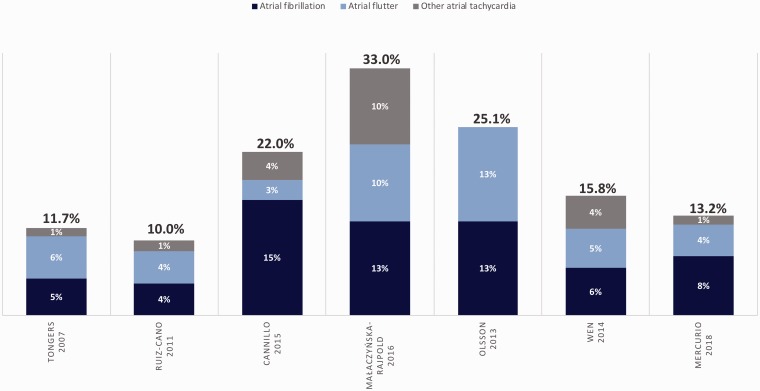
Overall frequency and subtypes of SVA (by study) in PAH/CTEPH. Subtypes: atrial fibrillation, atrial flutter, or other atrial tachycardia. SVA, supraventricular arrhythmia.

SVAs are the most prevalent rhythm disturbance observed in patients with PAH/CTEPH and they occur with greater incidence than in the general population.^1,70–72^ In this discussion of SVA, we are referring specifically to AF, AFl, atrioventricular nodal reentrant tachycardia (AVNRT), or atrial tachycardia, and not to sinus tachycardia. The onset of SVA often signals progressive right-sided cardiac dysfunction and frequently precipitates clinical decompensation by eliminating the atrial kick which is important to diastolic RV filling in the hypertrophied RV.^8,73,74^ Four retrospective and three prospective studies have examined the incidence and clinical relevance of SVA in PAH, in some cases including patients with CTEPH and in one study including patients with group 3 PH.^8,75–79^ The details of these studies are outlined in Tables 1 and 2. A graphical inter-study comparison of SVA frequency is depicted in Fig. 1. The seven studies are all small, with substantial limitations in methodology, but taken together, highlight six important points in regard to SVA and PAH/CTEPH.

**Table 1.** Retrospective studies of supraventricular arrhythmia (SVA) in PAH/CTEPH.

Baseline characteristics of study population								Incidence and outcomes					
First author (years), location	Study design	n (WHO group)	Subgroup	Age (years)*	Female (%)	6MWD (m)	mPAP (mmHg)* PCWP (mmHg)* Cardiac index (L/min/m^2^)*	Incidence of SVT (%)	Onset from PH diagnosis (months)	Type of SVA	Asym (%)^†^	Mortality in SVA group (%)	Mortality in permanent SVA (%)
Tongers (1998–2003), Germany	Single-center, retrospective cohort	231 (1,4)^‡^	IPAH 70% Group 4^‡^ 12% PAH-CTD 9% PPHTN 5% PAH-CHD 4% PAH-HIV 1%	48 ± 14	65	314 ± 128	54 ± 12 8 ± 3 2.1 ± 0.6	11.7	42 (0–238)	AF 42% AFl 48% AVNRT 10%	16	37	82^§^
Ruiz-Cano (1995–2008), Spain	Single-center, retrospective cohort	282 (1)	PAH-CTD 30% IPAH 26% PAH-DT 26% PAH-CHD 17%	47.3 ± 14.3	61	NR	NR	10	60 ± 56	AF 43% AFl 43% AVNRT 14%	18	22	NR
Cannillo (2008–2015), Italy	Single-center, retrospective cohort	77 (1,3,4)^‡^	PAH-CTD 23% IPAH 21% Group 4^‡^ 18% Group 3 16% PoPH 12% PAH-CHD 6% PAH-HIV 3% PAH-DT 1%	63 (48–70.7)	53	340 (188.7–428.7)	44 (35–54) NR 2.6 (2.2–3.4)	22	15 (11–43)	AF 70% AFl 12% Other** 17%	23	53	66^††^
Małaczyńska- Rajpold (2008–2013), Poland	Single-center, retrospective cohort	48 (1)	IPAH 63% PAH-CTD 21% PAH-CHD 17%	NR	69	NR	NR NR NR	33	NR	AF 38% AFl 31% ATach 31%	41	35	NR

^*^Mean ± SD or median (IQR).

^†^Patients asymptomatic at presentation with SVA (%).

^‡^Inoperable group 4 PH (CTEPH).

^§^*P* = 0.01, comparing permanent SVA group with SVA converted to sinus rhythm group.

^**^Included two atrial ectopic tachycardias and one AV nodal re-entry tachycardia.

^††^*P* = 0.001, comparing with no SVA cohort (13% mortality).

NR, not reported; 6MWD, 6-min walk distance; mPAP, mean pulmonary artery pressure; PCWP, pulmonary capillary wedge pressure; PH, pulmonary hypertension; IPAH, idiopathic pulmonary artery hypertension (PAH); PAH-CTD, connective tissue disease-related PAH; PPHTN, persistent pulmonary hypertension; PAH-CHD, congenital heart disease-related PAH; PAH-HIV, HIV-related PAH; PAH-DT, drug and toxin-related PAH; PoPH, portopulmonary hypertension; AF, atrial fibrillation; AFl, atrial flutter; AVNRT, AV nodal re-entrant tachycardia; ATach, atrial tachycardia.

**Table 2.** Prospective studies of supraventricular arrhythmia (SVA) in PAH/CTEPH.

Baseline characteristics of study population								Incidence and outcomes					
First author (years), location	Study design	n (WHO group)	Subgroup	Age (years)*	Female (%)	6MWD (m)*	mPAP (mmHg)* PCWP (mmHg)* Cardiac index (L/min/m^2^)*	Cumulative incidence of SVA	Type of SVA	Asym (%)^†^	HR for risk factors associated with SVA (95% CI)^‡,§^	HR for mortality in overall SVA group (95% CI)	HR for mortality in permanent SVA group (95% CI)
Olsson (2005–2010), Germany	Single-center, prospective cohort	239 (1,4)**	IPAH 39% Group 4** 34% PAH-CTD 11% PAH-CHD 9% PoPH 6% PAH-HIV 1%	55 (49–66)	61	335 (292–429)	47 (37–53) NR 2.5 (2.0–2.8)	13.4% (1st year) 19.2% (2nd year) 23.6% (3rd year) 25.1 % (5th year)	AF 50% AFl 50%	17	RAP^††^ 1.1 (1.1–1.2) mPAP^‡‡^ 1.0 (1.0–1.1) CI^§§^ 1.9 (1.1–3.5) NT-BNP*** 1.4 (1.1–1.9)	1.75 (1.1–3.0) *P* = 0.042	2.30 (1.3–6.0) *P* = 0.006
Wen (2007–2012), China	Multicenter, prospective cohort	280 (1)	IPAH 100%	39 ± 15	68	383 ± 95	62 ± 15 9 ± 5 2.5 ± 1.4	6.4% (1st year) 12.4% (3rd year) 15.8% (6th year)	AF 40% AFl 33% ATach 28%	2.5	RVD 2.4 (1.7–3.2) LAA 1.1 (1.0–1.2) mRAP 1.1 (1.1–1.1) PVR 1.1 (1.1–1.1)	2.15 (1.2–3.8) *P* < 0.001	3.79 (2.0–7.3) *P* < 0.001
Mercurio (2000–2016), USA	Single-center, prospective cohort	317 (1)	PAH-CTD^†††^ 63% IPAH 37%	57 ± 14	84	328 ± 129	45.5 ± 14 10.6 ± 4.0 4.4 ± 1.6 (CO, L/min)	13.2%	AF 60% AFl 32% ATach 9%	9.9	NR^‡‡‡^	NR^‡‡‡^	NR^‡‡‡^

^*^Mean ± SD or median (IQR).

^†^Patients asymptomatic at presentation with SVA (%).

^‡^Wen et al. hazard ratios reported after adjustment for covariates, including age and targeted therapy.

^§^*P* < 0.05.

^**^Inoperable group 4 PH (CTEPH).

^††^Per 1-mmHg increase.

^‡‡^Per 5-mmHg increase.

^§§^Per 0.5-L/min/m^2^ decrease.

^***^Per 50-ng/L increase.

^†††^All systemic sclerosis related-PAH.

^‡‡‡^HR not reported for these metrics. Patients with SVA had higher RAP (mmHg) (12.3 ± 6.1 vs. 8.9 ± 5.7; *P* = 0.002), PCWP (mmHg) (12.4 ± 3.8 vs. 10.3 ± 3.9; *P* = 0.002), and NT-proBNP (3168 ± 3398 vs. 1866 ± 3711; *P* = 0.046). Higher overall mortality in SVA group but not statistically significant (69% vs. 51%; log rank *P* = 0.323). Mortality HR not reported for permanent vs. transient SVA.

NR, not reported; 6MWD, 6-min walk distance; mPAP, mean pulmonary artery pressure; PCWP, pulmonary capillary wedge pressure; HR, hazard ratio; IPAH, idiopathic pulmonary artery hypertension (PAH); PAH-CTD, connective tissue disease-related PAH; PAH-CHD, congenital heart disease-related PAH; PAH-HIV, HIV-related PAH; PAH-DT, drug and toxin-related PAH; PoPH, portopulmonary hypertension; AF, atrial fibrillation; AFl, atrial flutter; AVNRT, AV nodal re-entrant tachycardia; SVA, supraventricular tachycardia; CI, cardiac index; NT-proBNP, NT-BNP, N-terminal pro b-type natriuretic peptide; RAP, right atrial pressure; LAA, left atrial area; mRAP, mean right atrial pressure; PVR, pulmonary vascular resistance; RVD, right ventricle diameter; CO, cardiac output.

### 1. There is no clear difference in baseline characteristics predicting SVA susceptibility

Baseline characteristics do not reliably predict which patients will develop SVA (Tables 1 and 2). However, the findings most correlated with development of SVA include elevated right atrial pressure (RAP)^75,76,78,80^ and markers of overall cardiac dysfunction, such as decreased cardiac index/output, increased RV diameter, and elevated NT-proBNP.^76,78,80^ Only in a minority of studies were pulmonary vascular resistance (PVR) and mPAP associated with incident SVA,^76,78^ perhaps suggesting it is the adaptation of the heart to increased afterload, rather than the afterload itself, that predicts development of SVA. There is biologic plausibility to this finding, given that the maladaptive response of the right heart is thought to predispose to atrial arrhythmias.^29,30^

Differences in non-invasive measures of disease severity, such as 6-minute walk distance (6MWD) and World Health Organization (WHO) functional class (FC), did not predict development of SVA in any of the studies. This is unexpected but may be a consequence of the overall homogeneity of the included cohorts, which largely included patients with poor functional state, or the relatively small sample size with less sensitive and more subjective measures.

Likewise, in most studies, the baseline PAH therapy and co-morbidities were no different between PAH/CTEPH patients with SVA versus those without. An exception is the study by Mercurio et al., who found a significantly increased frequency of thyroid disease (defined as hypo- or hyperthyroidism, or radiographic thyroid nodules) in the SVA group compared to those without SVA (69% vs. 43%).^80^ The clinical significance of thyroid disease, which is prevalent in PAH, was not explored in the other studies and may be an underrecognized contributing factor to development of arrhythmia.^81^

### 2. SVA often precipitates clinical decompensation

Onset of SVA often correlates with a clinically relevant decompensation. Whether the SVA triggers the clinical decompensation or is the result of right heart failure is not always clear; however, in many cases, clinical decompensation onsets abruptly with an episode of SVA and conversely resolves with restoration of sinus rhythm. The majority of patients reported symptoms at time of SVA onset (59–98%). Across all studies, objective and standardized measures of disease severity consistently worsened after development of SVA. The reported measures included worsening of New York Heart Association (NYHA) FC and a decrease in 6MWD. One study reported an increase in PAH-specific therapy in 46% of patients with SVA,^82^ while another noted that arrhythmia led to admission to the intensive care unit and vasopressor use in approximately 30% of SVA episodes.^80^

### 3. Some SVAs are subclinical and detection may require a screening ECG or ambulatory monitor

While the majority of patients do have symptoms at SVA onset (mean ∼80%), up to 41% of episodes are asymptomatic, with their arrhythmia identified only via screening ECG or ambulatory monitor. With the finding that SVA is associated with increased mortality in patients with PAH/CTEPH, the role for surveillance of PAH/CTEPH patients by means of routine ECG, Holter monitors, or loop recorders warrants further study.

### 4. Restoration of sinus rhythm reverses decompensation

Restoration of sinus rhythm coincides with the reversal of adverse effects of an episode of SVA. Atrioventricular synchrony in sinus rhythm increases cardiac output and is particularly important when there is diastolic dysfunction. Thus, it is likely the active, synchronous atrial loading of the ventricles that results from sinus rhythm increases cardiac output in PAH patients. Restoring sinus rhythm should therefore be strongly considered in patients with PH. However, as will be discussed in the management section, there is a lack of randomized clinical trial data comparing rate versus rhythm control in PAH/CTEPH patients. When reported, restoration of sinus rhythm was associated with objectively confirmed clinical improvement. For example, in one study, at the time of SVA onset, 6MWD decreased from 362 ± 114 m to 258 ± 147 m, returning to a value near baseline (345 ± 137 m) 6–12 weeks after restoration of sinus rhythm. Over the same time period, NT-proBNP decreased from 5926 ± 4648 ng/L at the time of SVA onset to 3360 ± 2804 ng/L after correction.^76^

### 5. Permanent SVA is associated with increased risk of death

**Fig. 2.**
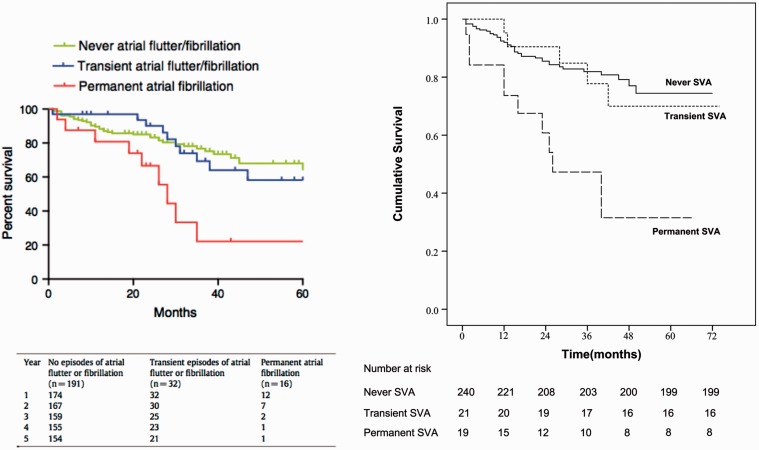
Comparative survival of PAH patients with and without SVA. Kaplan–Meier survival analyses from two prospective studies^76,78^ examining SVA in PAH. In both studies, permanent SVA was associated with increased mortality compared to transient SVA and never SVA. Included with permission.

SVA is associated with increased mortality, driven primarily by permanent (as compared to transient) SVA. In the four retrospective studies, mortality was in the range of 22–53% in the SVA cohort compared to 0–13% in those patients without SVA.^10,75,79,82^ Similarly, two of the prospective studies report elevated hazard ratios (HR) for mortality in those who develop SVA (HR = 1.75–2.15).^76,78^ A third study describes an increased mortality of 69% in the SVA group compared to 51% in those without SVA, although this was not statistically significant on survival analysis.^80^ The effect of permanent SVA on mortality is quite striking, as depicted in the survival curves in Fig. 2. The increase in mortality was almost entirely attributable to development of permanent SVA (HR = 2.3–3.8), as compared to transient or no SVA.^76,78^

### 6. Atrial fibrillation is the most common cause of permanent SVA in PAH/CTEPH

The most common cause of permanent SVA is AF (accounting for 42–70% of all cases). However, Afl is also prevalent, occurring in 12–50% of the series reviewed (Fig. 1). Less common SVAs include AVNRT and atrial tachycardia. The high incidence of AF has clinical implications given that restoration of sinus rhythm is more difficult in AF compared to other SVA, and that permanent SVA is associated with increased mortality. Clinicians should regard the development of permanent AF as a serious adverse event in a patient with PAH/CTEPH. Addition of permanent SVA to contemporary mortality prediction models (for example, REVEAL registry risk assessment) may help with prognostication, as it appears to portend a significant decline in survival.^1,83–86^

Additional large-scale investigations are needed to prospectively characterize the incidence of SVA in PH. While onset of SVA appears to be associated with increased mortality, it is not clear if the SVA is causal of deterioration or simply a result of worsening clinical condition. Unfortunately, given the nature of the disease process and the invasiveness required to obtain hemodynamic data, the frequent monitoring required to address this question may require these data be obtained as a part of large clinical trials or international registries. To date, the incidence/prevalence of SVA have not been reported in prospective or large-scale studies.^2,85,87,88^

### Management

#### Guidelines

There is currently no standardized guidance regarding management of SVA in PAH/CTEPH, although the options include both pharmacologic and non-pharmacologic interventions (Table 3). Given the improvement in hemodynamics and functional status observed with restoration of sinus rhythm,^10,76,78,79,82^ the ESC/ERS guidelines state that “although prospective and controlled data are lacking, [these] findings suggest that maintenance of a stable sinus rhythm after cardioversion should be considered an important treatment goal in patients with PAH.”^1^ The ESC/ERS guidelines also state that SVAs “represent an indication for oral anticoagulation” for stroke prevention, although the risk of stroke in PAH patients with Afl is unclear.^1^ In the absence of prospective and controlled data, no level of recommendation or strength of evidence for maintaining sinus rhythm or anticoagulation is provided. Furthermore, the 2009 American College of Cardiology Foundation (ACCF)/American Heart Association (AHA) expert consensus document on PH does explicitly address SVA management.^89^

**Table 3.** Recommendations for the management of SVAs in PAH and CTEPH.

1. Rhythm control, as opposed to rate control, should be considered as initial first-line therapy for SVA in PAH/CTEPH.
2. Pharmacologic rhythm control options include amiodarone, sotalol, dronedarone, and class 1C antiarrhythmics; however, the risk of drug–drug interaction with PAH/CTEPH medications should be carefully evaluated.
3. The use of short acting beta-blockers or calcium channel blockers for rate control in acute SVA can be considered with caution in the patient with acute decompensated right ventricular failure (RVF). The long-term use of beta-blocker or CCB therapy to maintain rate control in RVF requires further research.
4. The use of electrophysiology studies with catheter ablation appears to be a safe alternative to pharmacologic therapy, especially in those patients with atrial flutter.
5. The safety and efficacy of left atrial interventions, such as pulmonary vein isolation or the “maze” procedure, have not been studied in PAH/CTEPH.
6. In patients with PAH, anticoagulation can be considered, especially in those patients with permanent AF. However, the risks and benefits should be carefully weighed, as there is some evidence for harm with anticoagulant use certain subgroups of PAH (associated-PAH) and the current risk stratification models for anticoagulant use in atrial fibrillation (i.e. CHA2DS2-VASc) may not accurately represent this population.

#### Rate and rhythm control

Several studies have reported treatment practice patterns, highlighting the heterogeneity in real-world approach and outcomes.^10,75,76,78,79,82^ In three of the seven studies, 100% of patients with SVA had initial attempts at rhythm control with direct current cardioversion, radiofrequency ablation, or antiarrhythmic drug therapy.^10,76,78^ A rate control strategy was utilized in a minority of patients, typically in those with permanent AF.^75,79^ Rhythm control strategies were more successful in atrial flutter (46.2–100%) versus atrial fibrillation (16.7–67.0%). The treatment tactics used and their efficacy are outlined in Table 4, with the exception of the Ruiz-Cano study, which was excluded due to lack of detail regarding arrhythmia management.^82^ However, Ruiz-Cano did report that patients with AVNRT (which they called INRT) underwent catheter ablation of the slow pathway while those with typical Afl had cavotricuspid isthmus ablation. The management of AF was not discussed and outcomes were simply noted to be “similar to that of the general population.”^82^

**Table 4.** Management strategies and success rates of therapy for SVA in PAH/CTEPH.

	DCCV (%)	ABL (%)	ODP (%)	Pharmacologic	Sinus rhythm obtained	Recurrence of SVA (%)
Tongers (2007)	29.6	29.6	11.1	3.7% (digoxin)	59.3 (AF 16.7%; AFl 100%)	NR
Canillo (2015)	65.1	23.1	–	76.9% (sotalol, amiodarone, 1C antiarrhythmic)	65.0 (NR)	52.9
Małaczyńska-Rajpold (2016)	18.8	25.0	6.3	25.0% (amiodarone, propafenone)	68.8 (AF 50.0%; AFl 60.0%)	56.3
Olsson (2012)	56.3	33.3	2.1	85.4% (amiodarone, dronedarone, flecainide, digitalis)	77.1 (AF 67.0%; AFl 88.0%)	27.1
Wen (2014)	5.0	2.5	–	92.5% (amiodarone, digoxin)	52.5 (AF 25.0%; AFl 46.2%)	15.0
Mercurio (2018)	31.0	31.0	–	NR* (amiodarone, digoxin, metoprolol/ carvedilol, verapamil/ diltiazem, flecainide)	NR	64.3

* Overall percentage of patients receiving pharmacologic therapy not reported; some patients received more than one agent. Individual percentages of drug use are as follows: amiodarone 45%, digoxin 57%, metoprolol/carvedilol 43%, verapamil/diltiazem 43%, flecainide 2%.

DCCV, direct current cardioversion; ABL, ablation; ODP, overdrive pacing; AF, atrial fibrillation; AFl, atrial flutter; SVA, supraventricular arrhythmia.

A variety of pharmacologic strategies were used for SVA (Table 4) including digoxin, amiodarone, sotalol, dronedarone, and class 1C antiarrhythmic agents (flecainide, propafenone). Medical therapy for SVA in PAH/CTEPH is limited by concern for drug–drug interactions with antiarrhythmics and PAH-specific therapies, as well as adverse effects. For example, bosentan (an endothelin receptor antagonist [ERA]) is an inducer of CYP3A4 and may reduce levels of amiodarone or dronedarone.^90,91^ Many antiarrhythmic drugs have antihypertensive effects based on mechanism of action and may cause systemic hypotension when used concomitantly with phosphodiesterase inhibitors (sildenafil, tadalafil) or prostacyclins (epoprostenol).^90^ The use of beta-adrenergic blockers in PH is also controversial. There is risk of provoking heart failure or circulatory collapse, based on their well-established negative inotropic and chronotropic effects.^92,93^ More contemporary data have started to study the safety and efficacy of beta-blockers in patients with PAH who are free from congestion/heart failure,^94–96^ but the use of beta-blockers for improvement of RV function and/or survival remains a research question and is not standard of care. In contrast, the use of short acting beta-blockers may be helpful in restoring sinus rhythm or regulating heart rate in patients with acute SVA.

Amiodarone (both intravenous and oral administration) was the most frequently reported antiarrhythmic agent for medical cardioversion and/or maintenance of sinus rhythm. While amiodarone is typically well-tolerated in the short term, the long-term effects may compound morbidity in patients with PAH/CTEPH.^97^ In the studies of SVA in PAH/CTEPH, AFl and other atrial arrhythmias, such as AVNRT, were more readily converted to and maintained in sinus rhythm with amiodarone in short- and intermediate-term follow-up compared to AF.^10,76,79,82^

Electrophysiology studies with catheter ablation are safe, feasible, and successful in patients with PH.^98–102^ In a retrospective analysis of 22 patients with AFl and PAH/CTEPH, Showkathali et al. showed that AFl ablation at the cavotricuspid isthmus was successfully performed without complications in all patients and was associated with a statistically significant improvement in FC.^98^ The relative success of this interventional strategy can be attributed to the right-sided location of typical flutter or re-entrant pathways, allowing avoidance of trans-septal puncture thereby reducing procedural risks of stroke and bleeding.

Little is known about left heart interventions in PAH/CTEPH patients and it is unclear whether, or to what degree, left-sided pathways contribute to arrhythmia in this cohort. Were pulmonary veins and left atrial pathways to prove important in PAH/CTEPH-related SVA, caution would be required in terms of catheter-based therapeutics. There is uncertainty regarding the effect of pulmonary vein manipulation on pre-capillary pulmonary pressure.^75,79^ Pulmonary vein isolation is more invasive compared to catheter-based interventions in the right heart and concerns exist regarding the risk of anesthesia and intubation in patients with PAH/CTEPH. In general, intubation should be avoided in PAH patients due to the problematic effect of sedation on cardiac function and vasodilation leading to hemodynamic collapse.^103^ In-hospital mortality in PAH patients requiring vasopressor and mechanical ventilation for RV failure approaches 100%,^104^ justifiably increasing hesitancy to perform more invasive (and potentially low-yield) ablation procedures.

#### Anticoagulation

Since the use of anticoagulation in CTEPH is routine management, anticoagulation for SVA would not alter therapy; however, there is increasing recognition of the absence of benefit and potential for harm with anticoagulation in some forms of PAH.^105,106^ This highlights the value of more carefully establishing the risks and benefits of anticoagulation for arrhythmia in PAH patients.

In recent guidelines, oral anticoagulation is considered harmful in patients with associated-PAH but is recommended in idiopathic, heritable, or anorexigen-induced PAH (expert consensus^89^/Grade IIb^1^). It is uncertain whether these recommendations should change in the event of permanent atrial fibrillation and it is unknown if the current risk stratification models (such as CHA2DS2-VASc) are valid in PAH/CTEPH.^107^ Only the prospective studies by Olsson et al., Wen et al., and Mercurio et al. specifically comment on the frequency of anticoagulation therapy in the examined PAH/CTEPH patients: Olsson et al., 92% overall on oral anticoagulants;^76^ Wen et al., 73% in SVA cohort, overall use not reported;^78^ and Mercurio et al., 36% of SVA cohort, overall use not reported.^80^ These differences likely reflect international practice pattern variation and inclusion criteria, e.g. the Olsson study included CTEPH while the others did not. There is no apparent difference in risk of developing SVA or subsequent morbidity or mortality after onset of arrhythmia based on the use of anticoagulation;^76,78^ however, vascular outcomes such as stroke were not specifically addressed. Unfortunately, neither of the recent publications that examined the utility of anticoagulation in PAH (COMPERA and REVEAL) comment on atrial arrhythmias,^87,108^ the presence of which might alter the benefits of anticoagulation. With up to 41% of patients experiencing asymptomatic atrial arrhythmias (Tables 1 and 2), a study assessing incremental benefit in morbidity with anticoagulation would require screening ECG or ambulatory monitoring to capture all potentially relevant SVAs.

## Ventricular arrhythmia

Ventricular tachyarrhythmias (VT or VF) are thought to cause the majority of SCDs in adult patients.^109^ Registry data demonstrate that most PAH patients die from progressive right heart failure or SCD.^3,110^ Since SCD often occurs outside the hospital, the cause of sudden death in PAH patients has not been well elucidated. The basis of SCD is hypothesized to include arrhythmia, circulatory collapse, compression of left main coronary artery, or pulmonary artery rupture or dissection.^111^ In contrast to advanced LV failure, malignant ventricular arrhythmias are not frequently observed in PAH, despite plausible pathophysiologic changes in the ventricular tissue (fibrosis) and QTc prolongation. VF has been demonstrated in a monocrotaline-induced PAH rat model,^41^ but clinical investigations have only identified VT/VF in a minority of PAH patients with SCD (8%).^641^

Non-sustained ventricular tachycardia (NSVT) was recently identified to be more prevalent than previously thought in a cohort of non-group 2 PH patients.^112^ Nonetheless, in a follow-up study, NSVT was not correlated with increased mortality in PAH/CTEPH.^113^ Since routine and sustained rhythm monitoring in PAH/CTEPH is not a standard clinical practice, the possibility that VT/VF contributes to a higher proportion of SCD cannot be excluded.

### Antiarrhythmic and device therapy for SCD

There are currently no data to show that patients with PAH/CTEPH and RV dysfunction benefit from defibrillator implantation to prevent SCD. The guidelines and management for arrhythmia in left heart failure may not apply to RV failure, due to differences in the underlying disease processes. Just as medications that are beneficial for group 1 PAH have been shown to be harmful or ineffective in group 2 PH (and vice versa), strategies to prevent and treat SCD may also differ. Practice guidelines for prevention of SCD in PAH state that “antiarrhythmic therapy is not indicated for prevention of SCD in patients with PAH,” and, further, that patients may be at “high risk during surgical procedures, such as ICD implantation.”^111^ ICD implantation is often offered to non-PH patients who have a cardiac arrest secondary to documented VT or VF,^111,114^ but clinical judgment must be employed regarding use of ICDs or prophylactic antiarrhythmic agents in PAH/CTEPH, given the absence of any clinical trials demonstrating benefit in this cohort.

Likewise, the role of cardiac resynchronization therapy (CRT) in right heart failure due to PAH/CTEPH remains undefined.^115,116^ Ventricular dyssynchrony is observed in progressive RV failure associated with PAH, often visualized as paradoxical septal movement caused by RV pressure/volume overload that compresses the left ventricle.^117,118^ This dyssynchrony is thought to hinder LV diastolic filling resulting in decreased LV stroke volume.^119^ RV free wall pacing with resynchronization improves RV function in a computer simulation model of PAH, an animal model of PAH, and clinically in a small cohort of patients with CTEPH.^120–122^ However, further investigation is needed to confirm whether CRT is a valid therapeutic strategy in PAH/CTEPH patients with RV failure. Of note, in left heart failure, CRT has not been shown to be beneficial in patients with right bundle branch block or with QRS intervals of <160 ms, two features that are common in patients with RV failure from PH. This raises some skepticism about the potential role CRT in PAH/CTEPH.

## Conclusion

**Fig. 3.**
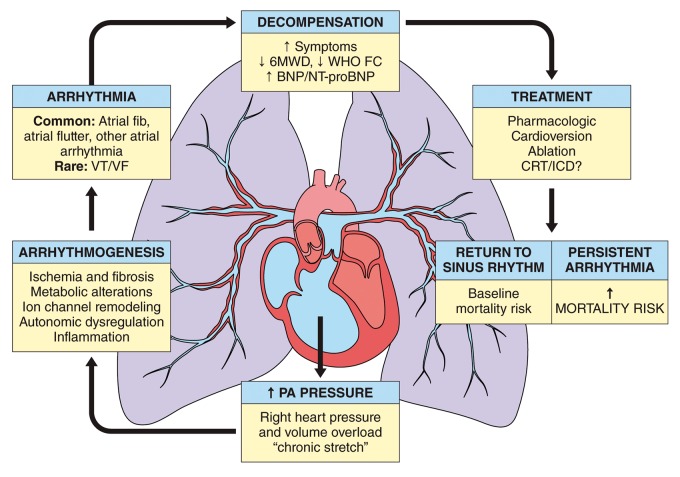
Schematic illustration outlining the development and consequence of arrhythmia in PH. CRT, cardiac resynchronization therapy; ICD, implanted cardiac defibrillator; VT, ventricular tachycardia; VF, ventricular fibrillation; fib, fibrillation; 6WMD, 6-min walk distance; WHO FC, World Health Organization functional class; BNP, brain natriuretic peptide; NT-proBNP, N-terminus-pro brain natriuretic peptide.

In summary, arrhythmias are an increasingly recognized cause of morbidity and mortality in patients with PAH/CTEPH.^123^ Despite ongoing research, there remain numerous gaps in knowledge, which limit a clinician's ability to optimally identify and manage arrhythmia in PAH/CTEPH (Table 5). SVAs, including AF and Afl, are more common than ventricular arrhythmias and, if sustained, portend clinical decompensation and increased mortality (Fig. 3). Improved screening and standardized management of SVA has the potential to improve outcomes in PAH patients. Randomized trials assessing rhythm versus rate control strategies and evaluating the role of anticoagulation are needed. Ventricular arrhythmias, while rare, can cause SCD in PAH/CTEPH. In contrast to left heart failure, Grade I evidence is lacking regarding the value of ICDs for primary prevention of such arrhythmias in PAH with RV failure. A large-scale surveillance study for sudden death etiology, perhaps using PH registries, could provide needed insight on the magnitude of arrhythmias and help to guide further recommendations for prevention and management.

**Table 5.** Opportunities for further investigation in arrhythmia and pulmonary hypertension.

Is there a role for surveillance monitoring with serial ECGs or continuous recording (such as with Holter monitor or loop recorder) to better understand the frequency of arrhythmia (supraventricular and ventricular) in PAH/CTEPH?
Why are ventricular tachycardia and ventricular fibrillation, which are prevalent in left-sided heart failure, not well characterized in the failing right ventricle of patients with PH?
Would markers of RV failure, such as serial measurements of brain natriuretic peptide (BNP), be predictive of the onset of arrhythmias? Biomarker surveillance may allow better understanding of the role of SVA as a cause versus a consequence of RV failure.
Which treatment strategy is superior (rhythm or rate control) for SVA in PAH/CTEPH?
What is the success of pharmacologic vs. non-pharmacologic treatment of SVA in PAH/CTEPH patients and what are the predictors of successful restoration of sinus rhythm?
Does the relatively high incidence of asymptomatic SVA in PAH/CTEPH lead to an increased incidence of stroke?
What is the role of anticoagulation in PAH/CTEPH patients with and without confirmed SVA, given the increased frequency of atrial arrhythmias in this population?
Is there a role for ICD placement in RV failure secondary to PAH/CTEPH?
Does cardiac resynchronization therapy provide benefit in RV failure secondary to PAH/CTEPH?

ECG, electrocardiogram; RV, right ventricular; ICD, implantable cardiac defibrillator; SVA, supraventricular arrhythmia.

## References

[1] Galiè N Humbert M Vachiery J-L et al. 2015 ESC/ERS Guidelines for the diagnosis and treatment of pulmonary hypertension. Eur Heart J 2015; 37: 67–119. 26320113 10.1093/eurheartj/ehv317

[2] Gall H Felix JF Schneck FK et al. The Giessen Pulmonary Hypertension Registry: Survival in pulmonary hypertension subgroups. J Heart Lung Transplant 2017; 36: 957–967. 28302503 10.1016/j.healun.2017.02.016

[3] D'Alonzo GE Barst RJ Ayres SM et al. Survival in patients with primary pulmonary hypertension. Results from a National Prospective Registry. Ann Intern Med 1991; 115: 343–349. 1863023 10.7326/0003-4819-115-5-343

[4] Wijeratne DT Lajkosz K Brogly SB et al. Increasing incidence and prevalence of World Health Organization Groups 1 to 4 pulmonary hypertension. Circ Cardiovasc Qual Outcomes 2018; 11: e003973. 29444925 10.1161/CIRCOUTCOMES.117.003973 PMC5819352

[5] Demerouti EA Manginas AN Athanassopoulos GD et al. Complications leading to sudden cardiac death in pulmonary arterial hypertension. Respir Care 2013; 58: 1246–1254. 23271814 10.4187/respcare.02252

[6] Hoeper MM Galié N Murali S et al. Outcome after cardiopulmonary resuscitation in patients with pulmonary arterial hypertension. Am J Respir Crit Care Med 2002; 165: 341–344. 11818318 10.1164/ajrccm.165.3.200109-0130c

[7] Tonelli AR Arelli V Minai OA et al. Causes and circumstances of death in pulmonary arterial hypertension. Am J Respir Crit Care Med 2013; 188: 365–369. 23600433 10.1164/rccm.201209-1640OC PMC3778730

[8] Rajdev A Garan H Biviano A Arrhythmias in pulmonary arterial hypertension. Prog Cardiovasc Dis 2012; 55: 180–186. 23009914 10.1016/j.pcad.2012.06.002 PMC3832144

[9] Anand V Roy SS Archer SL et al. Trends and outcomes of pulmonary arterial hypertension-related hospitalizations in the United States: analysis of the Nationwide Inpatient Sample Database from 2001 through 2012. JAMA Cardiol 2016; 1: 1021–1029. 27851838 10.1001/jamacardio.2016.3591 PMC12167389

[10] Tongers J Schwerdtfeger B Klein G et al. Incidence and clinical relevance of supraventricular tachyarrhythmias in pulmonary hypertension. Am Heart J 2007; 153: 127–132. 17174650 10.1016/j.ahj.2006.09.008

[11] Kanemoto N Sasamoto H Arrhythmias in primary pulmonary hypertension. Jpn Heart J 1979; 20: 765–775. 522240 10.1536/ihj.20.765

[12] James TN On the cause of syncope and sudden death in primary pulmonary hypertension. Ann Intern Med 1962; 56: 252–264. 14451032 10.7326/0003-4819-56-2-252

[13] Vaillancourt M Chia P Sarji S et al. Autonomic nervous system involvement in pulmonary arterial hypertension. Respir Res 2017; 18: 201. 29202826 10.1186/s12931-017-0679-6 PMC5715548

[14] Franciosi S Perry FKG Roston TM et al. The role of the autonomic nervous system in arrhythmias and sudden cardiac death. Auton Neurosci 2017; 205: 1–11. 28392310 10.1016/j.autneu.2017.03.005

[15] Ciarka A Doan V Velez-Roa S et al. Prognostic significance of sympathetic nervous system activation in pulmonary arterial hypertension. Am J Respir Crit Care Med 2010; 181: 1269–1275. 20194810 10.1164/rccm.200912-1856OC

[16] Velez-Roa S Ciarka A Najem B et al. Increased sympathetic nerve activity in pulmonary artery hypertension. Circulation 2004; 110: 1308–1312. 15337703 10.1161/01.CIR.0000140724.90898.D3

[17] Wensel R Jilek C Dorr M et al. Impaired cardiac autonomic control relates to disease severity in pulmonary hypertension. Eur Respir J 2009; 34: 895–901. 19443531 10.1183/09031936.00145708

[18] Folino AF Bobbo F Schiraldi C et al. Ventricular arrhythmias and autonomic profile in patients with primary pulmonary hypertension. Lung 2003; 181: 321–328. 14749936 10.1007/s00408-003-1034-x

[19] Carrio I Cowie MR Yamazaki J et al. Cardiac sympathetic imaging with mIBG in heart failure. JACC Cardiovasc Imaging 2010; 3: 92–100. 20129538 10.1016/j.jcmg.2009.07.014

[20] Cohen-Solal A Esanu Y Logeart D et al. Cardiac metaiodobenzylguanidine uptake in patients with moderate chronic heart failure: relationship with peak oxygen uptake and prognosis. J Am Coll Cardiol 1999; 33: 759–766. 10080478 10.1016/s0735-1097(98)00608-1

[21] Morimitsu T Miyahara Y Sinboku H et al. Iodine-123-metaiodobenzylguanidine myocardial imaging in patients with right ventricular pressure overload. J Nucl Med 1996; 37: 1343–1346. 8708768

[22] Sakamaki F Satoh T Nagaya N et al. Correlation between severity of pulmonary arterial hypertension and 123I-metaiodobenzylguanidine left ventricular imaging. J Nucl Med 2000; 41: 1127–1133. 10914900

[23] Maron BA Leopold JA Emerging concepts in the molecular basis of pulmonary arterial hypertension: part II: Neurohormonal signaling contributes to the pulmonary vascular and right ventricular pathophenotype of pulmonary arterial hypertension. Circulation 2015; 131: 2079–2091. 26056345 10.1161/CIRCULATIONAHA.114.006980 PMC4465126

[24] Ryan JJ Archer SL The right ventricle in pulmonary arterial hypertension: Disorders of metabolism, angiogenesis and adrenergic signaling in right ventricular failure. Circ Res 2014; 115: 176–188. 24951766 10.1161/CIRCRESAHA.113.301129 PMC4112290

[25] Ryan JJ Huston J Kutty S et al. Right ventricular adaptation and failure in pulmonary arterial hypertension. Can J Cardiol 2015; 31: 391–406. 25840092 10.1016/j.cjca.2015.01.023 PMC4385216

[26] Piao L Fang Y-H Parikh KS et al. GRK2-mediated inhibition of adrenergic and dopaminergic signaling in right ventricular hypertrophy: therapeutic implications in pulmonary hypertension. Circulation 2012; 126: 2859–2869. 23124027 10.1161/CIRCULATIONAHA.112.109868 PMC4459732

[27] Bristow MR Minobe W Rasmussen R et al. Beta-adrenergic neuroeffector abnormalities in the failing human heart are produced by local rather than systemic mechanisms. J Clin Invest 1992; 89: 803–815. 1311717 10.1172/JCI115659 PMC442925

[28] Nootens M Kaufmann E Rector T et al. Neurohormonal activation in patients with right ventricular failure from pulmonary hypertension: relation to hemodynamic variables and endothelin levels. J Am Coll Cardiol 1995; 26: 1581–1585. 7594089 10.1016/0735-1097(95)00399-1

[29] Zhao Q Deng H Jiang X et al. Effects of intrinsic and extrinsic cardiac nerves on atrial arrhythmia in experimental pulmonary artery hypertension. Hypertension 2015; 66: 1042–1049. 26418021 10.1161/HYPERTENSIONAHA.115.05846

[30] Medi C Kalman JM Ling L-HH et al. Atrial electrical and structural remodeling associated with longstanding pulmonary hypertension and right ventricular hypertrophy in humans. J Cardiovasc Electrophysiol 2012; 23: 614–620. 22269035 10.1111/j.1540-8167.2011.02255.x

[31] John B Stiles MK Kuklik P et al. Electrical remodelling of the left and right atria due to rheumatic mitral stenosis. Eur Heart J 2008; 29: 2234–2243. 18621772 10.1093/eurheartj/ehn329

[32] Sanders P Morton JB Davidson NC et al. Electrical remodeling of the atria in congestive heart failure: electrophysiological and electroanatomic mapping in humans. Circulation 2003; 108: 1461–1468. 12952837 10.1161/01.CIR.0000090688.49283.67

[33] Morton JB Sanders P Vohra JK et al. Effect of chronic right atrial stretch on atrial electrical remodeling in patients with an atrial septal defect. Circulation 2003; 107: 1775–1782. 12665497 10.1161/01.CIR.0000058164.68127.F2

[34] Medi C Kalman JM Spence SJ et al. Atrial electrical and structural changes associated with longstanding hypertension in humans: implications for the substrate for atrial fibrillation. J Cardiovasc Electrophysiol 2011; 22: 1317–1324. 21736657 10.1111/j.1540-8167.2011.02125.x

[35] Oral H Chugh A Good E et al. A tailored approach to catheter ablation of paroxysmal atrial fibrillation. Circulation 2006; 113: 1824–1831. 16606789 10.1161/CIRCULATIONAHA.105.601898

[36] Nattel S Burstein B Dobrev D Atrial remodeling and atrial fibrillation: mechanisms and implications. Circ Arrhythm Electrophysiol 2008; 1: 62–73. 19808395 10.1161/CIRCEP.107.754564

[37] Ozawa K Funabashi N Kataoka A et al. Myocardial fibrosis in the right ventricle detected on ECG gated 320 slice CT showed a short term poor prognosis in subjects with pulmonary hypertension. Int J Cardiol 2013; 168: 584–586. 23541610 10.1016/j.ijcard.2013.01.251

[38] Piao L Fang Y-H Cadete VJJ et al. The inhibition of pyruvate dehydrogenase kinase improves impaired cardiac function and electrical remodeling in two models of right ventricular hypertrophy: resuscitating the hibernating right ventricle. J Mol Med (Berl) 2010; 88: 47–60. 19949938 10.1007/s00109-009-0524-6 PMC3155251

[39] Fischer R Dechend R Gapelyuk A et al. Angiotensin II-induced sudden arrhythmic death and electrical remodeling. Am J Physiol Heart Circ Physiol 2007; 293: H1242–1253. 17416596 10.1152/ajpheart.01400.2006

[40] Tanaka Y Takase B Yao T et al. Right ventricular electrical remodeling and arrhythmogenic substrate in rat pulmonary hypertension. Am J Respir Cell Mol Biol 2013; 49: 426–436. 23600532 10.1165/rcmb.2012-0089OC

[41] Umar S Lee J-H de Lange E et al. Spontaneous ventricular fibrillation in right ventricular failure secondary to chronic pulmonary hypertension. Circ Arrhythm Electrophysiol 2012; 5: 181–190. 22199010 10.1161/CIRCEP.111.967265 PMC3319685

[42] Benoist D Stones R Drinkhill M et al. Arrhythmogenic substrate in hearts of rats with monocrotaline-induced pulmonary hypertension and right ventricular hypertrophy. Am J Physiol Heart Circ Physiol 2011; 300: H2230–2237. 21398591 10.1152/ajpheart.01226.2010 PMC3119089

[43] Benoist D Stones R Drinkhill MJ et al. Cardiac arrhythmia mechanisms in rats with heart failure induced by pulmonary hypertension. Am J Physiol Heart Circ Physiol 2012; 302: H2381–2395. 22427523 10.1152/ajpheart.01084.2011 PMC3378302

[44] Keldermann RH ten Tusscher KHWJ Nash MP et al. Effect of heterogeneous APD restitution on VF organization in a model of the human ventricles. Am J Physiol Heart Circ Physiol 2008; 294: H764–774. 18055526 10.1152/ajpheart.00906.2007

[45] Wickenden AD Kaprielian R Kassiri Z et al. The role of action potential prolongation and altered intracellular calcium handling in the pathogenesis of heart failure. Cardiovasc Res 1998; 37: 312–323. 9614488 10.1016/s0008-6363(97)00256-3

[46] Benoist D Stones R Benson AP et al. Systems approach to the study of stretch and arrhythmias in right ventricular failure induced in rats by monocrotaline. Prog Biophys Mol Biol 2014; 115: 162–172. 25016242 10.1016/j.pbiomolbio.2014.06.008 PMC4210667

[47] Sedlis SP Mechanisms of ventricular arrhythmias in acute ischemia and reperfusion. Cardiovasc Clin 1992; 22: 3–18. 1728431

[48] Luqman N Sung RJ Wang C-L et al. Myocardial ischemia and ventricular fibrillation: pathophysiology and clinical implications. Int J Cardiol 2007; 119: 283–290. 17166606 10.1016/j.ijcard.2006.09.016

[49] Torbicki A Kurzyna M Kuca P et al. Detectable serum cardiac troponin T as a marker of poor prognosis among patients with chronic precapillary pulmonary hypertension. Circulation 2003; 108: 844–848. 12900346 10.1161/01.CIR.0000084544.54513.E2

[50] van Wolferen SA Marcus JT Westerhof N et al. Right coronary artery flow impairment in patients with pulmonary hypertension. Eur Heart J 2008; 29: 120–127. 18065750 10.1093/eurheartj/ehm567

[51] Galiè N Saia F Palazzini M et al. Left main coronary artery compression in patients with pulmonary arterial hypertension and angina. J Am Coll Cardiol 2017; 69: 2808–2817. 28595696 10.1016/j.jacc.2017.03.597

[52] Bogaard HJ Abe K Vonk Noordegraaf A Voelkel NF The right ventricle under pressure: cellular and molecular mechanisms of right-heart failure in pulmonary hypertension. Chest 2009; 135: 794–804. 19265089 10.1378/chest.08-0492

[53] Bogaard HJ Natarajan R Henderson SC et al. Chronic pulmonary artery pressure elevation is insufficient to explain right heart failure. Circulation 2009; 120: 1951–1960. 19884466 10.1161/CIRCULATIONAHA.109.883843

[54] Archer SL Fang Y-H Ryan JJ Piao L Metabolism and bioenergetics in the right ventricle and pulmonary vasculature in pulmonary hypertension. Pulm Circ 2013; 3: 144–152. 23662191 10.4103/2045-8932.109960 PMC3641722

[55] Tonelli AR Baumgartner M Alkukhun L et al. Electrocardiography at diagnosis and close to the time of death in pulmonary arterial hypertension. Ann Noninvasive Electrocardiol 2014; 19: 258–265. 24372670 10.1111/anec.12125 PMC4004655

[56] Yi H-T Hsieh Y-C Wu T-J et al. Heart rate variability parameters and ventricular arrhythmia correlate with pulmonary arterial pressure in adult patients with idiopathic pulmonary arterial hypertension. Heart Lung 2014; 43: 534–540. 24929769 10.1016/j.hrtlng.2014.05.010

[57] Barr CS Naas A Freeman M et al. QT dispersion and sudden unexpected death in chronic heart failure. Lancet 1994; 343: 327–329. 7905146 10.1016/s0140-6736(94)91164-9

[58] Bluzaite I Brazdzionyte J Zaliūnas R et al. QT dispersion and heart rate variability in sudden death risk stratification in patients with ischemic heart disease. Medicina (Kaunas) 2006; 42: 450–454. 16816538

[59] Pye M Quinn AC Cobbe SM QT interval dispersion: a non-invasive marker of susceptibility to arrhythmia in patients with sustained ventricular arrhythmias?. Br Heart J 1994; 71: 511–514. 8043329 10.1136/hrt.71.6.511 PMC1025443

[60] Zareba W Moss AJ le Cessie S Dispersion of ventricular repolarization and arrhythmic cardiac death in coronary artery disease. Am J Cardiol 1994; 74: 550–553. 8074036 10.1016/0002-9149(94)90742-0

[61] Algra A Tijssen JG Roelandt JR et al. QT interval variables from 24 hour electrocardiography and the two year risk of sudden death. Br Heart J 1993; 70: 43–48. 8037997 10.1136/hrt.70.1.43 PMC1025227

[62] Algra A Tijssen JG Roelandt JR et al. QTc prolongation measured by standard 12-lead electrocardiography is an independent risk factor for sudden death due to cardiac arrest. Circulation 1991; 83: 1888–1894. 2040041 10.1161/01.cir.83.6.1888

[63] Malik M Batchvarov VN Measurement, interpretation and clinical potential of QT dispersion. J Am Coll Cardiol 2000; 36: 1749–1766. 11092641 10.1016/s0735-1097(00)00962-1

[64] Okin PM Devereux RB Howard BV et al. Assessment of QT interval and QT dispersion for prediction of all-cause and cardiovascular mortality in American Indians: The Strong Heart Study. Circulation 2000; 101: 61–66. 10618305 10.1161/01.cir.101.1.61

[65] Hong-liang Z Qin L Zhi-hong L et al. Heart rate-corrected QT interval and QT dispersion in patients with pulmonary hypertension. Wien Klin Wochenschr 2009; 121: 330–333. 19562296 10.1007/s00508-009-1184-9

[66] Rich JD Thenappan T Freed B et al. QTc prolongation is associated with impaired right ventricular function and predicts mortality in pulmonary hypertension. Int J Cardiol 2013; 167: 669–676. 22459397 10.1016/j.ijcard.2012.03.071 PMC3389574

[67] Zhou S Cao JM Tebb ZD et al. Modulation of QT interval by cardiac sympathetic nerve sprouting and the mechanisms of ventricular arrhythmia in a canine model of sudden cardiac death. J Cardiovasc Electrophysiol 2001; 12: 1068–1073. 11573698 10.1046/j.1540-8167.2001.01068.x

[68] Badagliacca R Reali M Poscia R et al. Right intraventricular dyssynchrony in idiopathic, heritable, and anorexigen-induced pulmonary arterial hypertension: clinical impact and reversibility. JACC Cardiovasc Imaging 2015; 8: 642–652. 25981504 10.1016/j.jcmg.2015.02.009

[69] Kalogeropoulos AP Georgiopoulou VV Howell S et al. Evaluation of right intraventricular dyssynchrony by two-dimensional strain echocardiography in patients with pulmonary arterial hypertension. J Am Soc Echocardiogr 2008; 21: 1028–1034. 18558476 10.1016/j.echo.2008.05.005

[70] Orejarena LA Vidaillet H DeStefano F et al. Paroxysmal supraventricular tachycardia in the general population. J Am Coll Cardiol 1998; 31: 150–157. 9426034 10.1016/s0735-1097(97)00422-1

[71] Heeringa J van der Kuip DAM Hofman A et al. Prevalence, incidence and lifetime risk of atrial fibrillation: the Rotterdam study. Eur Heart J 2006; 27: 949–953. 16527828 10.1093/eurheartj/ehi825

[72] Lloyd-Jones DM Wang TJ Leip EP et al. Lifetime risk for development of atrial fibrillation: the Framingham Heart Study. Circulation 2004; 110: 1042–1046. 15313941 10.1161/01.CIR.0000140263.20897.42

[73] Goldstein JA Harada A Yagi Y et al. Hemodynamic importance of systolic ventricular interaction, augmented right atrial contractility and atrioventricular synchrony in acute right ventricular dysfunction. J Am Coll Cardiol 1990; 16: 181–189. 2193048 10.1016/0735-1097(90)90477-7

[74] Gaine SP Naeije R Peacock AJ The Right Heart, London: Springer London, 2014.

[75] Małaczyńska-Rajpold K Komosa A Błaszyk K et al. The management of supraventricular tachyarrhythmias in patients with pulmonary arterial hypertension. Heart Lung Circ 2016; 25: 442–450. 26643289 10.1016/j.hlc.2015.10.008

[76] Olsson KM Nickel NP Tongers J et al. Atrial flutter and fibrillation in patients with pulmonary hypertension. Int J Cardiol 2013; 167: 2300–2305. 22727973 10.1016/j.ijcard.2012.06.024

[77] Rottlaender D Motloch LJ Schmidt D et al. Clinical impact of atrial fibrillation in patients with pulmonary hypertension. PLoS One 2012; 7: e33902. 22439013 10.1371/journal.pone.0033902 PMC3306317

[78] Wen L Sun M-L An P et al. Frequency of supraventricular arrhythmias in patients with idiopathic pulmonary arterial hypertension. Am J Cardiol 2014; 114: 1420–1425. 25217453 10.1016/j.amjcard.2014.07.079

[79] Cannillo M Grosso Marra W Gili S et al. Supraventricular arrhythmias in patients with pulmonary arterial hypertension. Am J Cardiol 2015; 116: 1883–1889. 26522342 10.1016/j.amjcard.2015.09.039

[80] Mercurio V Peloquin G Bourji KI et al. Pulmonary arterial hypertension and atrial arrhythmias: incidence, risk factors, and clinical impact. Pulm Circ 2018; 8: 2045894018769874. 29575972 10.1177/2045894018769874 PMC5912291

[81] Vakilian F Attaran D Shegofte M et al. Assessment of thyroid function in idiopathic pulmonary hypertension. Res Cardiovasc Med 2016; 5: e29361. 26949688 10.5812/cardiovascmed.29361 PMC4755060

[82] Ruiz-Cano MJ Gonzalez-Mansilla A Escribano P et al. Clinical implications of supraventricular arrhythmias in patients with severe pulmonary arterial hypertension. Int J Cardiol 2011; 146: 105–106. 21056484 10.1016/j.ijcard.2010.09.065

[83] Benza RL Gomberg-Maitland M Miller DP et al. The REVEAL registry risk score calculator in patients newly diagnosed with pulmonary arterial hypertension. Chest 2012; 141: 354–362. 21680644 10.1378/chest.11-0676

[84] Sitbon O Benza RL Badesch DB et al. Validation of two predictive models for survival in pulmonary arterial hypertension. Eur Respir J 2015; 46: 152–164. 25837032 10.1183/09031936.00004414

[85] Benza RL Miller DP Gomberg-Maitland M et al. Predicting survival in pulmonary arterial hypertension: insights from the Registry to Evaluate Early and Long-Term Pulmonary Arterial Hypertension Disease Management (REVEAL). Circulation 2010; 122: 164–172. 20585012 10.1161/CIRCULATIONAHA.109.898122

[86] Hoeper MM Kramer T Pan Z et al. Mortality in pulmonary arterial hypertension: prediction by the 2015 European pulmonary hypertension guidelines risk stratification model. Eur Respir J 2017; 50: 1700740. 28775047 10.1183/13993003.00740-2017

[87] Olsson KM Delcroix M Ghofrani HA et al. Anticoagulation and survival in pulmonary arterial hypertension: Results From the Comparative, Prospective Registry of Newly Initiated Therapies for Pulmonary Hypertension (COMPERA). Circulation 2014; 129: 57–65. 24081973 10.1161/CIRCULATIONAHA.113.004526

[88] Delcroix M Lang I Pepke-Zaba J et al. Long-term outcome of patients with chronic thromboembolic pulmonary hypertension: Results From an International Prospective Registry. Circulation 2016; 133: 859–871. 26826181 10.1161/CIRCULATIONAHA.115.016522

[89] McLaughlin VV Archer SL Badesch DB et al. ACCF/AHA 2009 Expert Consensus Document on Pulmonary Hypertension: A Report of the American College of Cardiology Foundation Task Force on Expert Consensus Documents and the American Heart Association: Developed in Collaboration With the American College. Circulation 2009; 119: 2250–2294. 19332472 10.1161/CIRCULATIONAHA.109.192230

[90] Anon. Lexi-Drugs. LexiComp Online.

[91] Venitz J Zack J Gillies H et al. Clinical pharmacokinetics and drug-drug interactions of endothelin receptor antagonists in pulmonary arterial hypertension. J Clin Pharmacol 2012; 52: 1784–1805. 22205719 10.1177/0091270011423662

[92] Peacock A Ross K Pulmonary hypertension: a contraindication to the use of {beta}-adrenoceptor blocking agents. Thorax 2010; 65: 454–455. 20435871 10.1136/thx.2008.111955

[93] Provencher S Herve P Jais X et al. Deleterious effects of β-blockers on exercise capacity and hemodynamics in patients with portopulmonary hypertension. Gastroenterology 2006; 130: 120–126. 16401475 10.1053/j.gastro.2005.10.013

[94] Thenappan T Roy SS Duval S et al. β-blocker therapy is not associated with adverse outcomes in patients with pulmonary arterial hypertension A propensity score analysis. Circ Heart Fail 2014; 7: 903–910. 25277998 10.1161/CIRCHEARTFAILURE.114.001429

[95] Bandyopadhyay D Bajaj NS Zein J et al. Outcomes of β-blocker use in pulmonary arterial hypertension: A propensity-matched analysis. Eur Respir J 2015; 46: 750–760. 26022959 10.1183/09031936.00215514

[96] Perros F De Man FS Bogaard HJ et al. Use of β-blockers in pulmonary hypertension. Circ Heart Fail 2017; 10: e003703. 28364092 10.1161/CIRCHEARTFAILURE.116.003703

[97] Siddoway LA Amiodarone: guidelines for use and monitoring. Am Fam Physician 2003; 68: 2189–2196. 14677664

[98] Showkathali R Tayebjee MH Grapsa J et al. Right atrial flutter isthmus ablation is feasible and results in acute clinical improvement in patients with persistent atrial flutter and severe pulmonary arterial hypertension. Int J Cardiol 2011; 149: 279–280. 21420184 10.1016/j.ijcard.2011.02.059

[99] Bandorski D Schmitt J Kurzlechner C et al. Electrophysiological studies in patients with pulmonary hypertension: a retrospective investigation. Biomed Res Int 2014; 2014: 617565. 24977152 10.1155/2014/617565 PMC4058223

[100] Bradfield J Shapiro S Finch W et al. Catheter ablation of typical atrial flutter in severe pulmonary hypertension. J Cardiovasc Electrophysiol 2012; 23: 1185–1190. 22734591 10.1111/j.1540-8167.2012.02387.x

[101] Garlitski AC Mark Estes NA 3rd Ablation of atrial flutter in severe pulmonary hypertension: pushing the outside of the envelope. J Cardiovasc Electrophysiol 2012; 23: 1191–1192. 22823280 10.1111/j.1540-8167.2012.02401.x

[102] Luesebrink U Fischer D Gezgin F et al. Ablation of typical right atrial flutter in patients with pulmonary hypertension. Heart Lung Circ 2012; 21: 695–699. 22795737 10.1016/j.hlc.2012.06.005

[103] Hoeper MM Granton J Intensive care unit management of patients with severe pulmonary hypertension and right heart failure. Am J Respir Crit Care Med 2011; 184: 1114–1124. 21700906 10.1164/rccm.201104-0662CI

[104] Campo A Mathai SC Le Pavec J et al. Outcomes of hospitalisation for right heart failure in pulmonary arterial hypertension. Eur Respir J 2011; 38: 359–367. 21310884 10.1183/09031936.00148310

[105] Cirulis MM Ryan JJ Where do we go from here? Reappraising the data on anticoagulation in pulmonary arterial hypertension. J Thorac Dis 2016; 8: E298–304. 27162687 10.21037/jtd.2016.03.67 PMC4842822

[106] Thenappan T Ormiston ML Ryan JJ et al. Pulmonary arterial hypertension: pathogenesis and clinical management. BMJ 2018; 360: j5492. 29540357 10.1136/bmj.j5492 PMC6889979

[107] Lip GYH Nieuwlaat R Pisters R et al. Refining clinical risk stratification for predicting stroke and thromboembolism in atrial fibrillation using a novel risk factor-based approach: the euro heart survey on atrial fibrillation. Chest 2010; 137: 263–272. 19762550 10.1378/chest.09-1584

[108] Preston IR Roberts KE Miller DP et al. Effect of warfarin treatment on survival of patients with pulmonary arterial hypertension (PAH) in the Registry to Evaluate Early and Long-Term PAH Disease Management (REVEAL). Circulation 2015; 132: 2403–2411. 26510696 10.1161/CIRCULATIONAHA.115.018435 PMC4689180

[109] Katritsis DG Gersh BJ Camm AJ A clinical perspective on sudden cardiac death. Arrhythmia Electrophysiol Rev 2016; 5: 177–182. 10.15420/aer.2016:11:2 PMC5248660 28116082

[110] Humbert M [A critical analysis of survival in idiopathic pulmonary arterial hypertension]. Presse Med 2010; 39(Suppl 1): 1S41–45. 20732617 10.1016/S0755-4982(10)70006-3

[111] Zipes DP Camm AJ Borggrefe M et al. ACC/AHA/ESC 2006 Guidelines for Management of Patients With Ventricular Arrhythmias and the Prevention of Sudden Cardiac DeathA Report of the American College of Cardiology/American Heart Association Task Force and the European Society of Cardiology Commi. J Am Coll Cardiol 2006; 48: e247–e346. 16949478 10.1016/j.jacc.2006.07.010

[112] Bandorski D Erkapic D Stempfl J et al. Ventricular tachycardias in patients with pulmonary hypertension: an underestimated prevalence? A prospective clinical study. Herzschrittmacherther Elektrophysiol 2015; 26: 155–162. 26031512 10.1007/s00399-015-0364-8

[113] Bandorski D Bogossian H Stempfl J et al. Prognostic relevance of nonsustained ventricular tachycardia in patients with pulmonary hypertension. Biomed Res Int 2016; 2016: 1327265. 28090536 10.1155/2016/1327265 PMC5206408

[114] Priori SG Blomström-Lundqvist C Mazzanti A et al. 2015 ESC Guidelines for the management of patients with ventricular arrhythmias and the prevention of sudden cardiac death: The Task Force for the Management of Patients with Ventricular Arrhythmias and the Prevention of Sudden Cardiac Death of the European Society of Cardiology (ESC) endorsed by: Association for European Paediatric and Congenital Cardiology. Eur Heart J 2015; 36: 2793–2867. 26320108 10.1093/eurheartj/ehv316

[115] Handoko ML de Man FS Allaart CP et al. Perspectives on novel therapeutic strategies for right heart failure in pulmonary arterial hypertension: lessons from the left heart. Eur Respir Rev 2010; 19: 72–82. 20956170 10.1183/09059180.00007109 PMC9491638

[116] Rasmussen JT Thenappan T Benditt DG et al. Is cardiac resynchronization therapy for right ventricular failure in pulmonary arterial hypertension of benefit?. Pulm Circ 2014; 4: 552–559. 25610593 10.1086/678470 PMC4278617

[117] Roeleveld RJ Marcus JT Faes TJC et al. Interventricular septal configuration at mr imaging and pulmonary arterial pressure in pulmonary hypertension. Radiology 2005; 234: 710–717. 15634939 10.1148/radiol.2343040151

[118] Tanaka H Tei C Nakao S et al. Diastolic bulging of the interventricular septum toward the left ventricle. An echocardiographic manifestation of negative interventricular pressure gradient between left and right ventricles during diastole. Circulation 1980; 62: 558–563. 7398017 10.1161/01.cir.62.3.558

[119] Marcus JT Gan CT-J Zwanenburg JJM et al. Interventricular mechanical asynchrony in pulmonary arterial hypertension: left-to-right delay in peak shortening is related to right ventricular overload and left ventricular underfilling. J Am Coll Cardiol 2008; 51: 750–757. 18279740 10.1016/j.jacc.2007.10.041

[120] Handoko ML Lamberts RR Redout EM et al. Right ventricular pacing improves right heart function in experimental pulmonary arterial hypertension: a study in the isolated heart. Am J Physiol Heart Circ Physiol 2009; 297: H1752–1759. 19734361 10.1152/ajpheart.00555.2009

[121] Lumens J Arts T Broers B et al. Right ventricular free wall pacing improves cardiac pump function in severe pulmonary arterial hypertension: a computer simulation analysis. Am J Physiol Heart Circ Physiol 2009; 297: H2196–2205. 19837949 10.1152/ajpheart.00870.2009

[122] Hardziyenka M Surie S De Groot JR et al. Right ventricular pacing improves haemodynamics in right ventricular failure from pressure overload: An open observational proof-of-principle study in patients with chronic thromboembolic pulmonary hypertension. Europace 2011; 13: 1753–1759. 21784747 10.1093/europace/eur189

[123] Wanamaker B Cascino T McLaughlin V et al. Atrial arrhythmias in pulmonary hypertension: pathogenesis, prognosis and management. Arrhythmia Electrophysiol Rev 2018; 7: 43. 10.15420/aer.2018.3.2 PMC5889803 29636972

